# Optimization of table tennis target detection algorithm guided by multi-scale feature fusion of deep learning

**DOI:** 10.1038/s41598-024-51865-3

**Published:** 2024-01-16

**Authors:** Zhang Rong

**Affiliations:** Shaanxi Energy Institute, Xi’an, 71000 Shaanxi China

**Keywords:** Computer science, Information technology

## Abstract

This paper aims to propose a table tennis target detection (TD) method based on deep learning (DL) and multi-scale feature fusion (MFF) to improve the detection accuracy of the ball in table tennis competition, optimize the training process of athletes, and improve the technical level. In this paper, DL technology is used to improve the accuracy of table tennis TD through MFF guidance. Initially, based on the FAST Region-based Convolutional Neural Network (FAST R-CNN), the TD is carried out in the table tennis match. Then, through the method of MFF guidance, different levels of feature information are fused, which improves the accuracy of TD. Through the experimental verification on the test set, it is found that the mean Average Precision (mAP) value of the target detection algorithm (TDA) proposed here reaches 87.3%, which is obviously superior to other TDAs and has higher robustness. The DL TDA combined with the proposed MFF can be applied to various detection fields and can help the application of TD in real life.

## Introduction

Table tennis is a sport that is widely spread all over the world. Its competition process is fast, tense and high-tech, which requires athletes to respond quickly and accurately control the trajectory of the ball. In table tennis competition, it is very important to accurately detect the track of table tennis for improving the fairness and technical level of the competition^1,2^. Traditional target detection (TD) methods face many challenges in table tennis competition. Firstly, due to the high speed and irregular trajectory of table tennis, the traditional target tracking algorithm may face the problem of target tracking loss. For example, when the ball is moving at high speed, the traditional algorithm may not be able to capture the position of the ball in time, resulting in the interruption of target tracking. This has a negative impact on athletes' technical training, because they need to accurately track the position of the ball to react. Secondly, the change of illumination is a common problem in table tennis matches, especially in indoor competition venues. Traditional target detection algorithm (TDA) may be sensitive to illumination changes, leading to detection errors. For example, under strong light, the shadow and reflection of the ball may make it difficult for the algorithm to correctly identify the position of the ball. This kind of detection error may lead to wrong training feedback and technical evaluation, which affects the technical improvement of athletes. In addition, table tennis players show rich movements and complex postures in the competition. Traditional TDAs may not be able to deal with these changes effectively, especially when athletes perform rapid and continuous movements. For example, athletes may adopt different hitting postures, and traditional algorithms may produce false detection or missed detection due to the diversity of postures, which affects the accurate evaluation of technical actions in training and competition^3^. The training process of table tennis players also needs accurate target detection algorithm (TDA) to help them better master the technology^4^. For example, in the training process, it is necessary to record the player's hitting posture, the rotation angle and speed of the ball and other information. However, the accuracy of this information depends on the performance of TDA, so it is very important to use high-precision TDA for athletes' technical training^5^.

But with deep learning (DL) technology's ongoing advancement and use, this issue of TD is now effectively resolved. Convolutional Neural Network (CNN) has become one of the most widely used methods for picture TD in DL CNN. Compared with traditional image processing algorithms, CNN can automatically learn image features and identify and locate targets more accurately^6^. However, in table tennis, due to the high speed and complexity of table tennis, the problem of TD cannot be solved well by using single-scale features^7^. As a result, this paper proposes the DL approach and the multi-scale feature fusion (MFF) guiding method to enhance the precision of table tennis target identification and optimize the training process of players, thereby enhancing their technical proficiency and competition quality. First, the TD in the table tennis match is done using a Fast Region-Based Convolutional Neural Network (Fast R-CNN). Then, to increase the precision of TD, several layers of feature information are fused using the MFF guidance technique.

The following are the paper's contributions:

1. The paper proposes a FAST R-CNN-based MFF TD approach, which may be utilized to successfully improve the accuracy and robustness of TD in challenging moving settings.
2. Through DL technology, image features are automatically learned, which avoids the tedious process that traditional TD methods need to manually extract features.
3. The training process of table tennis players is optimized, which improves the effect and accuracy of technical training.

The main innovation of this paper is to propose a specific method combining FAST R-CNN and MFF technology. By introducing MFF into FAST R-CNN, this method not only realizes automatic feature extraction, but also optimizes MFF to improve the accuracy and robustness of target detection. As the basis of deep learning algorithm, FAST R-CNN realizes feature extraction of RoI with different sizes through RoI pooling layer, while MFF technology introduces FPN structure, which enables the network to better extract feature information at different scales. Therefore, the research innovation lies in improving the effect of table tennis target detection through the combination of deep learning of specific structure and MFF. But, this paper not conduct in-depth research on the optimization of table tennis technical training. Secondly, this paper only uses one dataset for experimental verification, and the dataset is relatively small, which lacks a comprehensive verification of the robustness of the algorithm. Finally, different algorithms may have different effects for different input resolutions, and may increase the calculation and memory occupation while improving the accuracy. In addition, although the introduction of FPN improves the performance of the algorithm, it is necessary to weigh the relationship between accuracy and reasoning time (Supplementary Information).

## Research status of TD based on MFF of DL

Regarding the use of various DL techniques for TD, Jiang et al. (2021) carried out real-time ship detection using the You Only Look Once version 4 (YOLO v4) TD method and fully utilized the multi-channel Synthetic Aperture Radar (SAR) image processing method based on image information and network feature extraction ability^8^. In their research on the identification and classification of road markings in conjunction with visible light camera sensors, Hoang et al. (2019) used the RetinaNet object detection approach. They also used Focal Loss to address the issue of class imbalance^9^. In their study on TD in self-driving automobiles, Li et al. (2022) used the CenterNet single-stage TD approach, which considerably simplified TD by employing the central point rather than the bounding box^10^. A lightweight underwater TD system based on YOLO v4 and MFF was proposed by Zhang et al. in 2021. This approach introduced the State Action Model (SAM) structure and the CSP Dark Net 53 (Cross Stage Partial Dark Net 53) network to achieve MFF while simultaneously utilizing a variety of data improvement techniques to increase the model's resilience and generalizability^11^. According to Wang et al. (2020), High-Resolution Network (HRNet) used high-resolution feature maps to fuse multi-scale data and used a multi-branch structure to increase detection accuracy^12^. Multiple Region-based Convolutional Neural Network (R-CNN) models were cascaded to execute MFF in the Cascade Region-based Convolutional Neural Network (Cascade R-CNN) that Cai et al. (2019) suggested. The detection accuracy was increased by bounding box refinement and hard negative mining^13^. Hou et al. (2022) used the transformer-based DNN to recognize human movements, and the overall classification rate of six human movements reached 94.96%, which provided high-precision recognition in real-time actual scenes^14^. Neupane et al. (2022) studied and fine-tuned You Only Look Once (YOLO) network, and proposed a multi-vehicle tracking algorithm, which can obtain the vehicle count, classification and speed of each lane in real time^15^. The research proved that the accuracy was doubled after fine-tuning. By comparing four YOLO networks, the You Only Look Once Version 5 Large (YOLOv5-large) network was combined with their tracking algorithm, which provided a trade-off between overall accuracy, loss and model size. Fu et al. (2023) proposed a DL method of field dependent deep learning localization (FD-Deep Loc), which was used to accurately locate the spatially variable point emission sources in the whole range of modern scientific Complementary Metal–Oxide–Semiconductor (sCMOS) camera chip^16^. Meimetis et al. (2023) combined Deep Simple Online and Real Time Tracking (Deep SORT) with YOLO detection method, and realized real-time multi-target tracking through concrete and multi-dimensional performance analysis in different traffic video datasets and various real-world materials, mainly for vehicles and pedestrians^17^. Li et al. (2023) proposed a small target depth convolution recognition algorithm based on the improved You Only Look Once version 4 (YOLOv4) network, and the feasibility of the algorithm was verified by using small electronic components to build a dataset on an industrial assembly line^18^. The experimental results showed that compared with the original YOLOv4, the average detection speed of the improved network was increased by about 30%, and the accuracy was improved by about 7%. Dong et al. (2023) proposed a multi-space residual network structure, which improved the performance of TD by introducing residual channel pooling and MFF structure, and achieved competitive results in the experiment^19^. Qi et al. (2022) integrated special feature extraction and information fusion technology and proposed a single-stage small object detection network, which effectively improved the performance of small TD^20^.

In conclusion, MFF technology has been extensively applied in DL-TD and has produced specific outcomes. Zhuang et al. (2019) proposed a MFF detector, which used a single-shot detection framework and a MFF module to detect on three different scales. It was found that the proposed method had excellent detection accuracy and computational efficiency^21^. To achieve real-time network intrusion detection, Zhang et al. (2020) coupled the support vector machine (SVM) method and deep belief network^22^. To increase the precision of TD, Liu et al. (2022) used an attention mechanism and an MFF network with a Convolutional NeXt (ConvNeXt) module^23^. According to the findings of the experiments, this approach is more accurate at detecting and locating more challenging items. A brand-new indoor style recognition technique employing multi-scale characteristics and lifting^24^ was put forth by Yaguchi et al. (2022). The results showed that the accuracy of the suggested strategy had increased by 0.021 compared to the residual network and by 0.128 compared to the conventional method. Dong et al. (2020) suggested a CNN approach based on balanced multi-scale fusion, and it was evaluated using an open Very High Resolution (VHR) remote sensing image set. Additionally, they discovered that the CNN method, which was based on balanced MFF, outperformed the existing standard detection techniques overall^25^.

However, there are still some shortcomings in the research of table tennis TDA: for fast moving objects in table tennis competition, the accuracy of MFF needs to be further improved. The influence of table tennis players' training process on the performance of TDA needs to be further studied. The MFF-based system still has a restricted scope of use for detecting targets in table tennis and needs further experimental verification. In light of the aforementioned shortcomings, the research proposes a DL MFF guidance-based table tennis TD approach. On the basis of FAST R-CNN, the MFF technique is presented to combine feature information from several levels to increase the accuracy of TD. Meanwhile, the performance of the algorithm is optimized, and player training effectiveness is enhanced through research into data gathering and data preprocessing in the training process of table tennis players. These achievements are of great significance for further studying the application of MFF technology in TD and promoting the development of athletes' training and competition.

## Overview of working model

**Figure 1:**
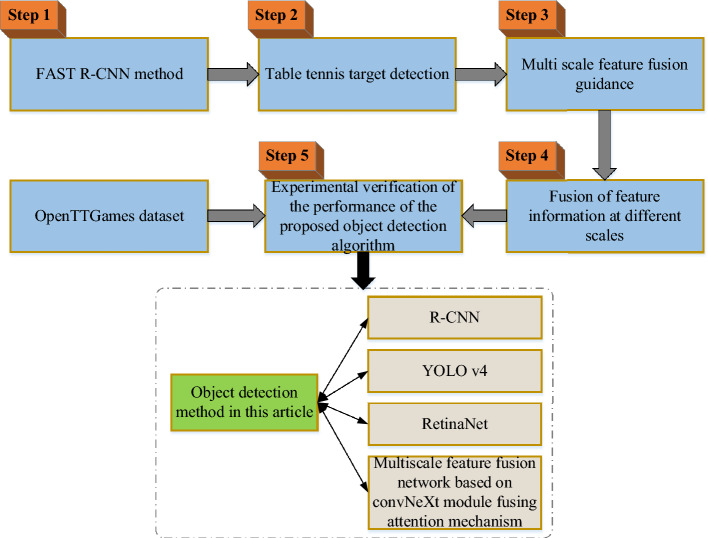
Workflow.

Firstly, the FAST R-CNN method is used to detect table tennis targets. Then, to increase the precision of TD, feature information from various scales is combined using the MFF guidance method. The TD approach in this paper is tested with the MFF network of R-CNN, YOLO v4, RetinaNet, and ConvNeXt module with attention mechanism, and the results are verified by experiments on the OpenTTGames dataset. The DL-based MFF guidance-based table tennis TD technique that was proposed in this paper has demonstrated outstanding performance in table tennis competition. In addition, this paper also applies the proposed method to the training process of athletes, which can help athletes better master the technology and improve the competition level. Figure 1 shows the workflow:

## Research methodology

### FAST R-CNN method

**Figure 2:**
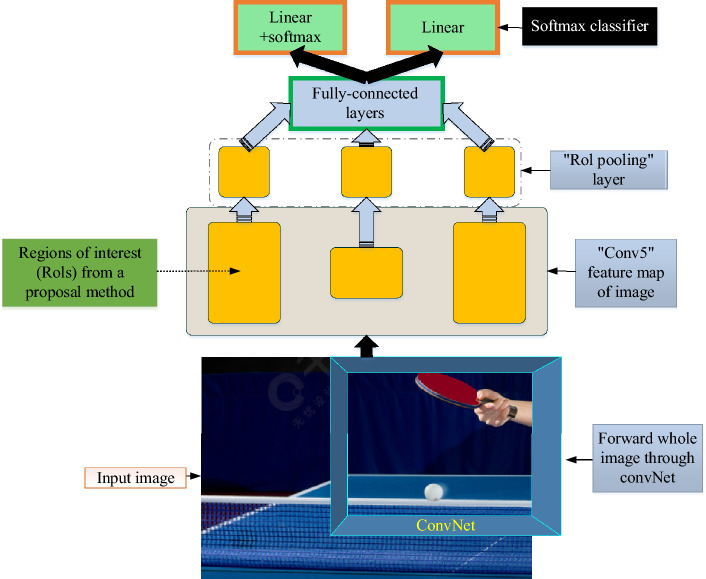
The network structure of Fast R-CNN.

In table tennis, the trajectory of table tennis is fast and complex, so the TDA needs to have high accuracy and robustness. Fast R-CNN is a DL algorithm for object recognition, which identifies and locates the target in the image by running CNN in the whole image. Compared with R-CNN and SPP-Net, Fast R-CNN has faster training and reasoning speed and higher accuracy^26^. Figure 2 shows the network architecture of the Fast R-CNN.

**Figure 3:**
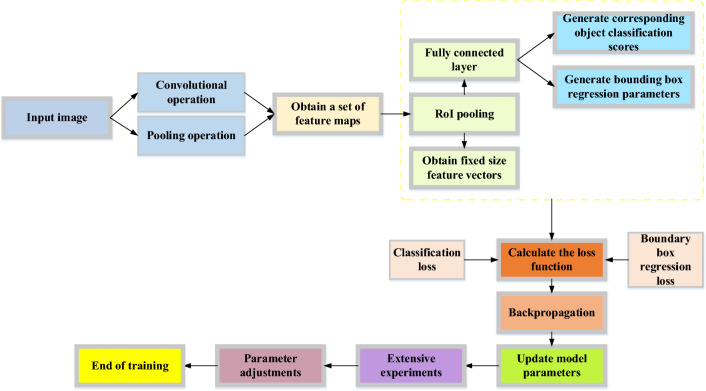
The training process of Fast R-CNN.

On the basis of the table tennis movement feature map, which is optimized using the original R-CNN model, Fig. 2 constructs a Fast R-CNN network structure. The RoI pool layer in the Fast R-CNN network can extract the same RoI resize with multiple sizes, which lessens the necessity for resizing RoI and accelerates detection. Fast R-CNN replaces the original SVM classifier with softmax classifier, which can directly estimate the probability of each category^27,28^. Figure 3 depicts the Fast R-CNN's training procedure.

In the network model training method depicted in Fig. 3, the Fast R-CNN network model is pre-trained using the ImageNet classification dataset. Additionally, Fast R-CNN must modify the original classification network's structure to complete the detection task. The first stage is to swap out the last pooling layer for a RoI pooling layer, where the grid's number of rows H and columns W must match the input scale of the layer below it that is the first fully connected layer. The second stage is to add two parallel fully connected layers in place of the final fully connected layer, one for classification and the other for getting back to the target frame's position. The third stage is splitting the network's input into two components: the image list and the RoI on those images^29^.

In the training process of Fast R-CNN, a fixed number of images, each containing a fixed number of RoI, can be used to calculate, which can improve the calculation speed and model convergence efficiency^30^. Table 1 illustrates the benefits of the FAST R-CNN approach over the conventional TDA:

**Table 1:** The benefits of the FAST R-CNN approach over the conventional TDA.

FAST R-CNN algorithm advantage	Explanation
End-to-end training	The manual feature extraction process is avoided, and the detection precision is increased
Region proposal network (RPN)	The quantity of candidate zones is decreased, and the speed of detection is increased
Shared convolution feature	The network as a whole share the convolution property, which lowers the number of calculations required and increases the speed of detection
Multitask loss function	the tasks of target classification and position regression are considered, which can improve the detection accuracy

The FAST R-CNN network has two parallel output branches. For each RoI, the first branch calculates the classification probabilities of $k$ target categories and 1 background category, $P=p=({p}_{0},\cdots ,{p}_{K})$. The second branch calculates the normalized offset and scaling ratio of the candidate frame^31^. The normalized offset and scaling ratio corresponding to the kth category are recorded as ${t}^{k}=({t}_{x}^{k},{t}_{y}^{k},{t}_{w}^{k},{t}_{h}^{k})$. For each RoI, the expression for calculating the joint loss of classification and location regression is shown in Eq. (1):

1 $${\text{L}}({\text{p,u}},t^{u} ,{\text{v}}) = L_{cls} (p,u) + \lambda [u \ge 1]L_{loc} (t^{u} ,v)$$

In Eq. (1), ${\text{p}}$ is the prediction probability of network classification, ${\text{u}}$ is the actual category, ${t}^{u}$ is the prediction boundary box, ${\text{v}}$ is the ground truth boundary box, and ${L}_{loc}({t}^{u},{\text{v}})$ represents the actual normalized offset of position loss to category $u$ and the normalized offset of scaling tuples ${u}_{x}$, ${u}_{y}$, ${u}_{w}$ and ${u}_{h}$ from the actual prediction and scaling tuples ${t}^{u}=({t}_{x}^{u},{t}_{y}^{u},{t}_{w}^{u},{t}_{h}^{u})$. ${L}_{cls}\left({\text{p}},{\text{u}}\right)$ represents the classification loss, and the calculation is shown in Eq. (2):

2 $${L}_{cls}\left(p,u\right)=-log{p}_{u}$$

In Eq. (2), ${p}_{u}$ is the probability that the network predicts that the RoI belongs to category $u$, and $u$ is the actual category label. The calculation of ${L}_{loc}({t}^{u},{\text{v}})$ is shown in Eqs. (3) and (4):

3 $${L}_{loc}({t}^{u},{\text{v}})=\sum_{i\in \left\{x,y,w,h\right\}}{smooth}_{{L}_{1}}({t}_{i}^{u}-{v}_{i})$$

4 $${smooth}_{{L}_{1}}(x)=\left\{\begin{array}{c}0.5{x}^{2},if\left|x\right|<1\\ \left|x\right|-0.5,otherwise\end{array}\right.$$

In the above equation, ${smooth}_{{L}_{1}}(x)$ is an indicative function. When $x=true$, $x=1$, otherwise $x=0$. When ${\text{u}}$ is the target category, $x=1$, and when ${\text{u}}$ is the background, $x=0$. ${t}_{i}^{u}$ is the *i*-th regression coefficient of prediction, ${v}_{i}$ is the $i$-th regression coefficient of ground truth, and $w$ and $h$ are the coefficients to punish the deviation of prediction. Equations (5) and (6) show the calculation of the $j$-th output after $r$ rois are maximized by the RoI pooling layer:

5 $${y}_{rj}={x}_{i*(r,j)}$$

6 $$i*(r,j)={argmax}_{{i}^{\mathrm{^{\prime}}}\in R(r,j)}{x}_{i}^{\mathrm{^{\prime}}}$$

In the above equation, ${i}{\prime}\in R(r,j)$ is the feature set of the corresponding index ${x}_{i}$ with ${y}_{rj}$ as the maximum pooled output, ${x}_{i}$ is the $i$th input of the pooled layer, ${x}_{i}{\prime}$ represents the corresponding index features of the pooled layer. $r$ RoI refers to the $r$ regions of interest detected in a picture, and the $j$th output refers to $r$ after processing. This value is obtained by maximizing the pool of the $j$th RoI. For the input of RoI layer, the partial derivative is shown in Eq. (7):

7 $$\frac{\partial L}{\partial {x}_{i}}=\sum_{r}\sum_{j}[i={i}^{*}\left(r,j\right)]\frac{\partial L}{\partial {y}_{rj}}$$

In Eq. (7), *L* represents the measurement method between different distances. For each RoI, the derivatives of its characteristic map will be assigned to different positions according to the pooling operation, and finally these derivatives will be summed to get the total derivatives of the input variables in the RoI pooling layer.

### MFF network structure

**Figure 4:**
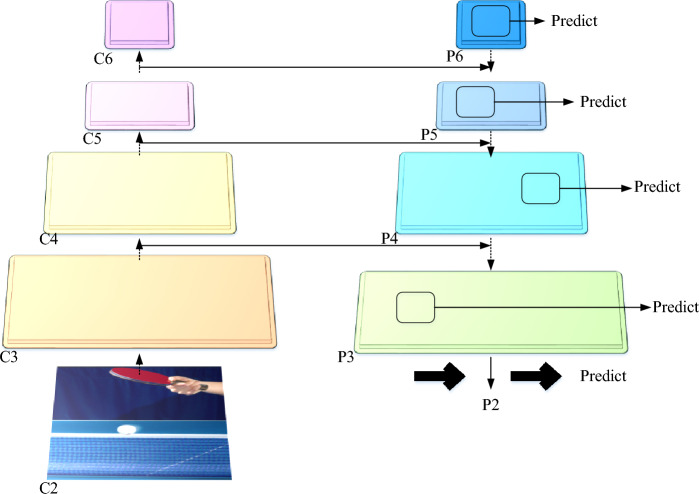
The network structure of FPN.

Feature extraction network and feature fusion (FF) network are the two main components of the MFF network construction. The feature extraction network typically uses a CNN structure, which allows for multi-layer convolution and pooling of the input image to extract the feature information at various scales. These feature maps may contain target information at several scales, but since each scale's feature map can only extract target information at that scale, it is required to combine the feature maps of other scales to acquire target information that is more complete^32^. FF networks usually adopt cross-scale FF methods, such as Feature Pyramid Network (FPN) and path aggregation network (PANet). These techniques enable the fusion of feature maps at various scales, which are then sent to the following classifiers and regressors for TD. To get the optimal TD impact, MFF network topology can be developed and modified in accordance with certain tasks and datasets^33^. The paper uses an MFF network based on FAST R-CNN and FPN structure to enhance TD's accuracy and stability in the task of table tennis. Figure 4 illustrates the FPN network structure.

The fundamental concept of FPN is to build a feature pyramid, where the high-level feature maps correspond to features with low resolution but strong semantic information, and the low-level feature maps correspond to features with high resolution but weak semantic information. To create feature pyramids with rich semantic information and high resolution, FPN fuses various tiers of feature maps. The FPN module is made up of two parts: the top-down path and the lateral connection. The high-level feature map is sampled using the top-down method down to the same resolution as the low-level feature map, and then the two feature maps are combined. To further enhance the semantic information of features, a horizontal connection is created between the high-level feature map and the low-level feature map^34,35^. Finally, FPN inputs the fused feature map into Fast R-CNN for detection. Equation (8) shows the expressions of FPN up sampling and fusion calculation:

8 $${P}_{n}=U\left({F}_{n}\right)+{F}_{n-1}$$

In Eq. (8), ${F}_{n}$ represents the feature map of the $n$th layer, and $U$ is an up-sampling operation. ${F}_{n}$ is up-sampled twice to make it the same size as ${F}_{n-1}$, and then added and fused to obtain a new feature map ${P}_{n}$. Equation (9) shows the multi-scale feature pyramid set:

9 $$P=\left\{{P}_{0},{P}_{1},{P}_{2},{P}_{3},{P}_{4}\right\}$$

In Eq. (9), ${{\text{P}}}_{0}$ is the initial feature map obtained by convolution of the input image, and ${{\text{P}}}_{0}$, ${{\text{P}}}_{2}$, ${{\text{P}}}_{3}$ and ${{\text{P}}}_{4}$ are multi-scale feature pyramids obtained by up-sampling and fusion of different scales. Equation (10) shows the top-down path expression:

10 $${P}_{n}{\prime}=\left\{\begin{array}{c}{P}_{n},ifn=N\\ U\left({P}_{n}\right)+{F}_{n-1}{\prime},otherwise\end{array}\right.$$

In Eq. (10), ${F}_{n-1}{\prime}$ is the feature map of the upper layer, and $U$ is the up-sampling operation, which upsamples ${P}_{n}$ twice, and then adds and fuses it with ${F}_{n-1}{\prime}$ to obtain a new feature map ${P}_{n}{\prime}$. Equation (11) shows the bottom-up path:

11 $${C}_{n}=\left\{\begin{array}{c}conv({F}_{n}),ifn=N\\ conv\left({F}_{n}\right)+U\left({C}_{n+1}\right),otherwise\end{array}\right.$$

In Eq. (11), $conv({F}_{n})$ is a convolution operation, and ${C}_{n}$ represents the feature information from the upper layer to the lower layer.

### TDA based on MFF of FAST-R-CNN

The DL approach for TD in table tennis suggested in this paper combines the FAST-R-CNN algorithm and the MFF technology. The introduction of MFF aims to improve the detection ability of the model and make it more robust by fusing the feature information of different scales to adapt to the fast and complex table tennis trajectory. The two main parts of the approach are TD and MFF. The FAST-R-CNN technique is first used to detect targets. As the basic framework of target detection, FAST R-CNN combines deep learning technology to improve the accuracy of the target, and enhances the robustness to complex scenes through deep feature learning. Using RoI pooling to extract characteristics from the region of interest. TD is performed by selectively searching the generated candidate regions. Combined with Feature Pyramid Network (FPN), features can be effectively extracted at different scales. The pyramid structure of FPN allows the network to focus on targets of different scales at different levels. This design can use fewer parameters to capture multi-scale information in the image, instead of introducing more parameters by increasing the depth of the network. One of the reasons for choosing FAST R-CNN as the basic network is that it performs well in TD tasks, and combined with FPN, it can better handle targets of different sizes. This combined network structure maintains the ability of accurate TD, but through the pyramid structure of FPN, it can avoid introducing too many redundant parameters. Therefore, the goal of structural design is to reduce the number of network parameters as much as possible under the premise of high accuracy to improve the efficiency of training and reasoning. By using feature pyramids of different scales, FPN can extract multi-scale features of images at different levels. This enables the network to capture the representation of targets in different sizes, thus improving the detection ability of small targets. Because of the pyramid structure of FPN, the network can better organize and utilize multi-scale features without introducing too many redundant parameters by increasing the depth of the network. The top-down and bottom-up information transmission mechanism of pyramid structure enables the network to make more effective use of semantic information on different scales, rather than simply introducing additional parameters by increasing the number of layers. To sum up, the structure design and feature fusion mechanism of this paper aims at reducing the number of network parameters to the maximum extent and improving the overall training and reasoning efficiency by choosing the appropriate network structure and feature fusion mode at the same time.

**Figure 5:**
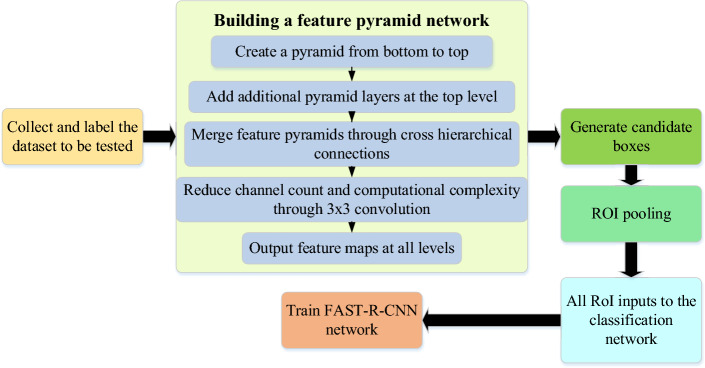
The flow chart of TDA combined with MFF of FAST-R-CNN.

In this paper, FAST-R-CNN algorithm is applied to the TD task in table tennis competition to detect and locate table tennis in the competition. Secondly, the accuracy of the detection is increased with the introduction of MFF technology. FPN algorithm is used to realize MFF. FPN algorithm can fuse features on different scales, thus extracting feature information on different scales to obtain better detection results. Specifically, FPN algorithm is composed of multiple sub-networks, and each sub-network processes input feature maps of different scales. Multi-layer feature maps are produced in each subnet using an up- and down-sampling framework, and several feature maps are combined by horizontal connection. Finally, the FAST-R-CNN algorithm is utilized to detect targets using the fused feature map. Figure 5 depicts the flowchart of the TDA when paired with the MFF of the FAST-R-CNN.

In Fig. 5, the target is detected in a single scale while the method is being built using the FAST-R-CNN algorithm. Then, the detection results and multi-scale feature maps are input into FPN algorithm for FF. Finally, the fused feature map is input into FAST-R-CNN algorithm again for TD, and the final detection result is obtained. Table 2 shows some codes of the TDA of FPN based on FAST-R-CNN:

**Table 2:** Some codes of the TDA of FPN based on FAST-R-CNN.

import keras backend as K
from keras import Model
from keras.layers import Input, Conv2D, MaxPooling2D, Flatten, Dense
def build_fpn(num_classes = 3):
input_image = Input (shape = (None, None, 3))
c1 = Conv2D(64, (3, 3), activation = 'relu', padding = 'same', name = 'block1_conv1')(input_image)
c1 = Conv2D(64, (3, 3), activation = 'relu', padding = 'same', name = 'block1_conv2')(c1)
p1 = MaxPooling2D((2, 2), strides = (2, 2), name = 'block1_pool')(c1)
c2 = Conv2D(128, (3, 3), activation = 'relu', padding = 'same', name = 'block2_conv1')(p1)
c2 = Conv2D(128, (3, 3), activation = 'relu', padding = 'same', name = 'block2_conv2')(c2)
p2 = MaxPooling2D((2, 2), strides = (2, 2), name = 'block2_pool')(c2)
c3 = Conv2D(256, (3, 3), activation = 'relu', padding = 'same', name = 'block3_conv1')(p2)
c3 = Conv2D(256, (3, 3), activation = 'relu', padding = 'same', name = 'block3_conv2')(c3)
c3 = Conv2D(256, (3, 3), activation = 'relu', padding = 'same', name = 'block3_conv3')(c3)
p3 = MaxPooling2D((2, 2), strides = (2, 2), name = 'block3_pool')(c3)
c4 = Conv2D(512, (3, 3), activation = 'relu', padding = 'same', name = 'block4_conv1')(p3)
c4 = Conv2D(512, (3, 3), activation = 'relu', padding = 'same', name = 'block4_conv2')(c4)
c4 = Conv2D(512, (3, 3), activation = 'relu', padding = 'same', name = 'block4_conv3')(c4)
p4 = MaxPooling2D((2, 2), strides = (2, 2), name = 'block4_pool')(c4)
c5 = Conv2D(512, (3, 3), activation = 'relu', padding = 'same', name = 'block5_conv1')(p4)
c5 = Conv2D(512, (3, 3), activation = 'relu', padding = 'same', name = 'block5_conv2')(c5)
c5 = Conv2D(512, (3, 3), activation = 'relu', padding = 'same', name = 'block5_conv3')(c5)
p5 = MaxPooling2D((2, 2), strides = (2, 2), name = 'block5_pool')(c5)
p6 = Conv2D(2048, (3, 3), strides = (2, 2), padding = 'same', activation = 'relu')(p5)
u5 = Conv2D(256, (1, 1), activation = 'relu', padding = 'same')(c5)
u5 = keras.layers.UpSampling2D(size = (2, 2))(u5)
u4 = Conv2D(256, (1, 1), activation = 'relu', padding = 'same')(c4)
u3 = Conv2D(256, (1, 1), activation = 'relu', padding = 'same')(c3)
u3 = keras.layers.UpSampling2D(size = (2, 2))(u3)
u2 = Conv2D(256, (1, 1), activation = 'relu', padding = 'same')(c2)
u2 = keras.layers.UpSampling2D(size = (4, 4))(u2)
merged = keras.layers.Concatenate(axis = 3)([u5, u4, u3, u2, p6])
final = Conv2D(256, (3, 3), padding = 'same', activation = 'relu')(merged)
input_rois = Input(shape = (None, 4))
roi_pool = ROI pooling([final, input_rois])
fc1 = Dense(4096, name = 'fc1')(roi_pool)
fc1 = keras.layers.Activation('relu')(fc1)
fc2 = Dense(4096, name = 'fc2')(fc1)
fc2 = keras.layers.Activation('relu')(fc2)
classifications = Dense(num_classes, activation = 'softmax', name = 'classifications')(fc2)
regressions = Dense(4 * num_classes, name = 'regressions')(fc2)
model = Model(inputs = [input_image, input_rois], outputs = [classifications, regressions])
return model

### Experimental preparation

The OpenTTGames dataset is used in the experiment in this paper. This dataset is a substantial TD dataset that includes 8000 photos with annotated frames. Among them, 4000 are used for training, 2000 for verification and 2000 for testing. Information about table tennis positions, sizes, and categories are contained in each label box. The position information of the label box has been provided in the dataset, which can be used to train and test the TDA. The dataset contains a variety of different scenes, such as singles, doubles, different perspectives, lighting conditions and the motion state of the ball. The public availability of this dataset can aid in the advancement of the study and use of the table tennis TDA. Source of dataset: OpenTTGames Dataset (osai.ai). The dataset includes full HD table tennis video recorded with industrial camera at 120 FPS. Each video is equipped with scene segmentation labels, including the labels of people, tables and scoreboards. The labeled image of the original dataset is a 320 × 128 color image, which needs to be converted into a gray labeled image with the same resolution as the video frame. Table 3 shows the code of reading data and converting data format.

**Table 3:** Code for reading data and converting data format.

import os
import cv2
import json
import tqdm
import numpy as np
def convert(video_name = 'game_1'):
seg_labels = ['Background', 'Player', 'Table', 'Scoreboard']
video_mp4 = f'{video_name}.mp4'
frame_dir = os.path.join(video_name, 'frames')
label_dir = os.path.join(video_name, 'labels')
mask_dir = os.path.join(video_name, 'segmentation_masks')
ball_json = os.path.join(video_name, 'ball_markup.json')
seg_label_txt = os.path.join(video_name, 'seg_label.txt')
seg_txt = os.path.join(video_name, 'seg.txt')
if not os.path.isdir(frame_dir):
os.mkdir(frame_dir)
if not os.path.isdir(label_dir):
os.mkdir(label_dir)
with open(seg_label_txt, 'w', encoding = 'UTF-8') as f:
for item in seg_labels:
f.write(f'{item}\n')
with open(ball_json, 'r', encoding = 'UTF-8') as f:
ball_markup = json.load(f)
frame_indexs = [int(item) for item in ball_markup.keys()]
video = cv2.VideoCapture(video_mp4)
seg_list = []
for index in tqdm.tqdm(frame_indexs):
frame_jpg = os.path.join(frame_dir, f'{index}.jpg')
mask_png = os.path.join(mask_dir, f'{index}.png')
label_png = os.path.join(label_dir, f'{index}.png')
mask = cv2.imread(mask_png)
mask[np.all(mask = = (0, 255, 0), axis = -1)] = (1, 1, 1)
mask[np.all(mask = = (0, 0, 255), axis = -1)] = (2, 2, 2)
mask[np.all(mask = = (0, 255, 255), axis = -1)] = (2, 2, 2)
mask[np.all(mask = = (255, 0, 0), axis = -1)] = (3, 3, 3)
mask[np.all(mask = = (255, 255, 0), axis = -1)] = (3, 3, 3)
label = cv2.resize(mask[…, 0], (1920, 1080), interpolation = 0)
video.set(cv2.CAP_PROP_POS_FRAMES, index)
res, frame = video.read()
cv2.imwrite(frame_jpg, frame)
cv2.imwrite(label_png, label)
seg_list.append(f'{frame_jpg} {label_png}')
with open(seg_txt, 'w', encoding = 'UTF-8') as f:
for item in seg_list:
f.write(f'{item}\n')
if __name__ = = '__main__':
data_dir = './dataset'
train_names = ['game_1']
val_names = ['test_1']
for name in train_names + val_names:
convert(os.path.join(data_dir, name))

The Windows 10 operating system serves as the experimental setting for this paper. The central processing unit (CPU), an Intel Core i7 7700 k, has 16 GB of memory. The graphics processing unit (GPU), a GTX 1080, has 6 GB of memory. TensorFlow 2.0 is adopted as a DL framework in this paper. TensorFlow is a powerful open-source DL framework developed by Google, which provides a wide range of tools and libraries to support various DL tasks, including TD. TensorFlow version 2.0 introduces functions such as Eager Execution and Keras integration, which makes the DL task more intuitive and easier to realize. Programming language: Python3.6. The OpenTTGames dataset is divided into training set, verification set and test set with a ratio of 2:1:1. This division is helpful to verify the model in the training process and evaluate its performance on the test set. In this model training procedure, the experimental model's iteration count is set to 300, the input image's resolution is 416*416 pixels, and the learning rate is 0.0001.

In this paper, the following TD evaluation indicators are used to measure the performance of FAST-R-CNN MFF TDA in table tennis TD^36,37^, and the specific expressions are shown in Eqs.(12–16):

12 $${\text{Accuracy}}={\text{TP}}/({\text{TP}}+{\text{FP}}+{\text{FN}})$$

13 $${\text{Precision}}={\text{TP}}/({\text{TP}}+{\text{FP}})$$

14 $${\text{Recall}}={\text{TP}}/({\text{TP}}+{\text{FN}})$$

15 $${\text{AP}}={\int }_{0}^{1}{\text{Precision}}({\text{Recall}}){\text{dRecall}}$$

16 $${\text{mAP}}=\frac{\sum_{i=1}^{n}A{P}_{i}}{n}$$

The number of targets that the algorithm accurately identified is represented by ${\text{TP}}$ in the equation above. The number of targets that the algorithm mistook for background or other objects is represented by ${\text{FP}}$. The number of targets that the algorithm misidentified is represented by ${\text{FN}}$. ${\text{AP}}$ stands for average precision, ${\text{mAP}}$ for average precision across multiple target categories, n for the number of target categories, and $A{P}_{i}$ for average precision across all of the target categories. The false detection rate of TD is given in Eqs. (17) and (18):

17 $$\mathcal{x}=\frac{FP}{FP+FN}$$

18 $${\text{MR}}={2}^{\left[{\text{log}}\left({x}_{1}\right)+{\text{log}}\left({x}_{2}\right)+\cdots +{\text{log}}\left({x}_{n}\right)\right]+n}$$

In the above equation, $\mathcal{x}$ represents the false detection of each image, and ${\text{MR}}$ represents the false detection of the target category^38^.

## Results and discussions

### Experimental results of different input image resolutions and different TDAs

The influence of different parameter settings on the performance of FAST R-CNN + FPN algorithm in this paper is shown in Table 4. In Table 4, in the process of table tennis TD in this paper, the parameter setting with learning rate of 0.0001 and iteration number of 300 is selected because it has achieved relatively good performance in mAP, accuracy and reasoning time.

**Table 4:** Different parameter settings affect the performance of FAST R-CNN + FPN algorithm in this paper.

Parameter setting	mAP (%)	Accuracy (%)	Reasoning time (ms)
Learning rate: 0.001, iteration times: 500	86.7	91.5	22
Learning rate: 0.0001, iteration times: 300	87.3	92.8	18
Learning rate: 0.0001, iteration times: 500	85.2	90.4	20

**Figure 6:**
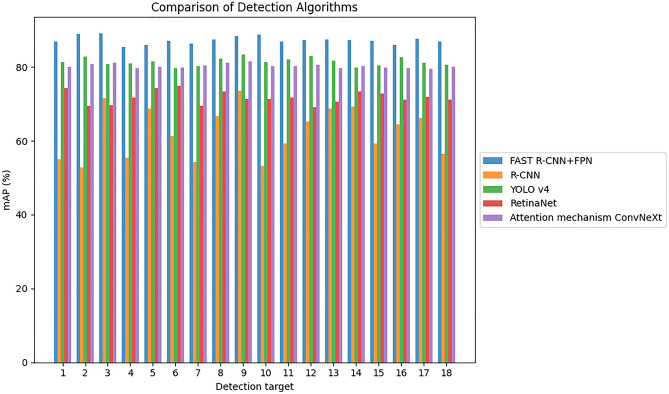
Experimental results of different TDAs.

The experimental outcomes of various target identification algorithms are displayed in Fig. 6 by contrasting the performance of the TD method in this paper with the TDAs of MFF networks of R-CNN, YOLO v4, RetinaNet, and ConvNeXt with attention mechanism. In Fig. 6, the mAP value of the FAST R-CNN + FPN method is shown to be greater than those of YOLO v4, Attention mechanism ConvNeXt, RetinaNet, and R-CNN, with an average mAP value of 87.30% for table tennis TD. The average mAP value of YOLO v4 algorithm in table tennis TD is 81.40%, and that of Attention mechanism ConvNeXt algorithm in table tennis TD is 80.3%. To sum up, FPN is introduced on the basis of FAST R-CNN, and multi-scale features are fused by top-down deconvolution, which further improves the performance of the algorithm. However, some algorithms, such as YOLO v4, Attention mechanism ConvNeXt and RetinaNet, are relatively simple in FF and do not make full use of feature information of different scales, so they perform relatively poorly in table tennis TD.

**Figure 7:**
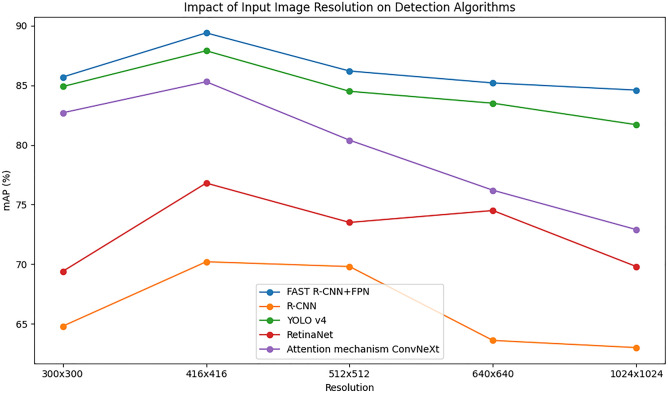
Influence of different input image resolutions on TDA.

Through experiments on OpenTTGames TD dataset, the effects of different input image resolutions on TDA are compared. In Fig. 7, the experimental findings are displayed.

In Fig. 7, the resolution of 416 × 416 input image is the best in table tennis TD, and the highest mAP value is 89.40%. However, with the increase of image resolution, the accuracy of different algorithms in table tennis TD shows a decreasing trend in different degrees. From the average results of TD mAP, the performance of 416 × 416 input image resolution in table tennis TD is better than that of 512 × 512 and 300 × 300. This result may be because in table tennis, the size of the ball is relatively small, and the high resolution of the input image may lead to too many pixels of the ball, which makes it impossible to effectively extract the feature information of the ball. In addition, using higher input resolution will increase the amount of calculation and memory occupation, thus affecting the speed and efficiency of the algorithm, and may even lead to over-fitting.

### Performance analysis of TDAs in different FF networks

**Figure 8:**
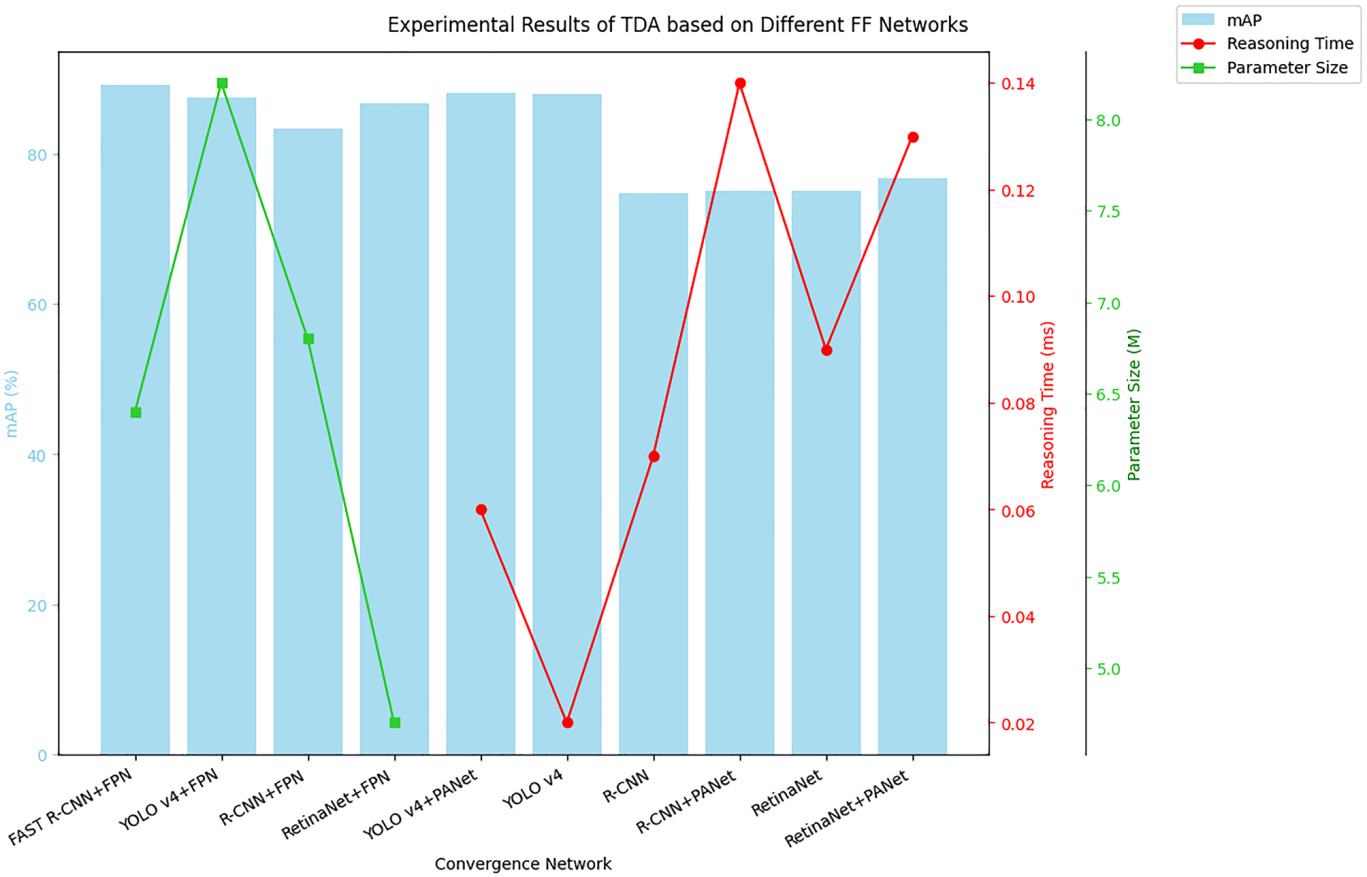
Experimental results of TDA based on different FF networks.

This section compares the effects of using different FF networks of FPN and PANet on different DL TDAs. Figure 8 displays the experimental outcomes of TDAs for various FF networks. In Fig. 8, using PANet to perform the DL TD task improves the accuracy, but the reasoning time also increases accordingly. Among them, the maximum mAP of YOLO v4 + PANet TDA can reach 88.10%, but the reasoning time is increased by 0.04 ms. The maximum mAP of FAST R-CNN + FPN proposed in this paper can reach 89.20%. Compared with other fusion methods, the accuracy is improved and the number of parameters is less. This is because the FAST R-CNN + FPN proposed in this paper adopts the simplified and efficient network structure of FAST R-CNN, combined with FPN, it can reduce the number of parameters with high accuracy, and FPN can reduce the redundancy of network parameters through MFF. However, other fusion networks with different characteristics of FPN and PANet have deep and multi-level network structure and more parameters.

**Figure 9:**
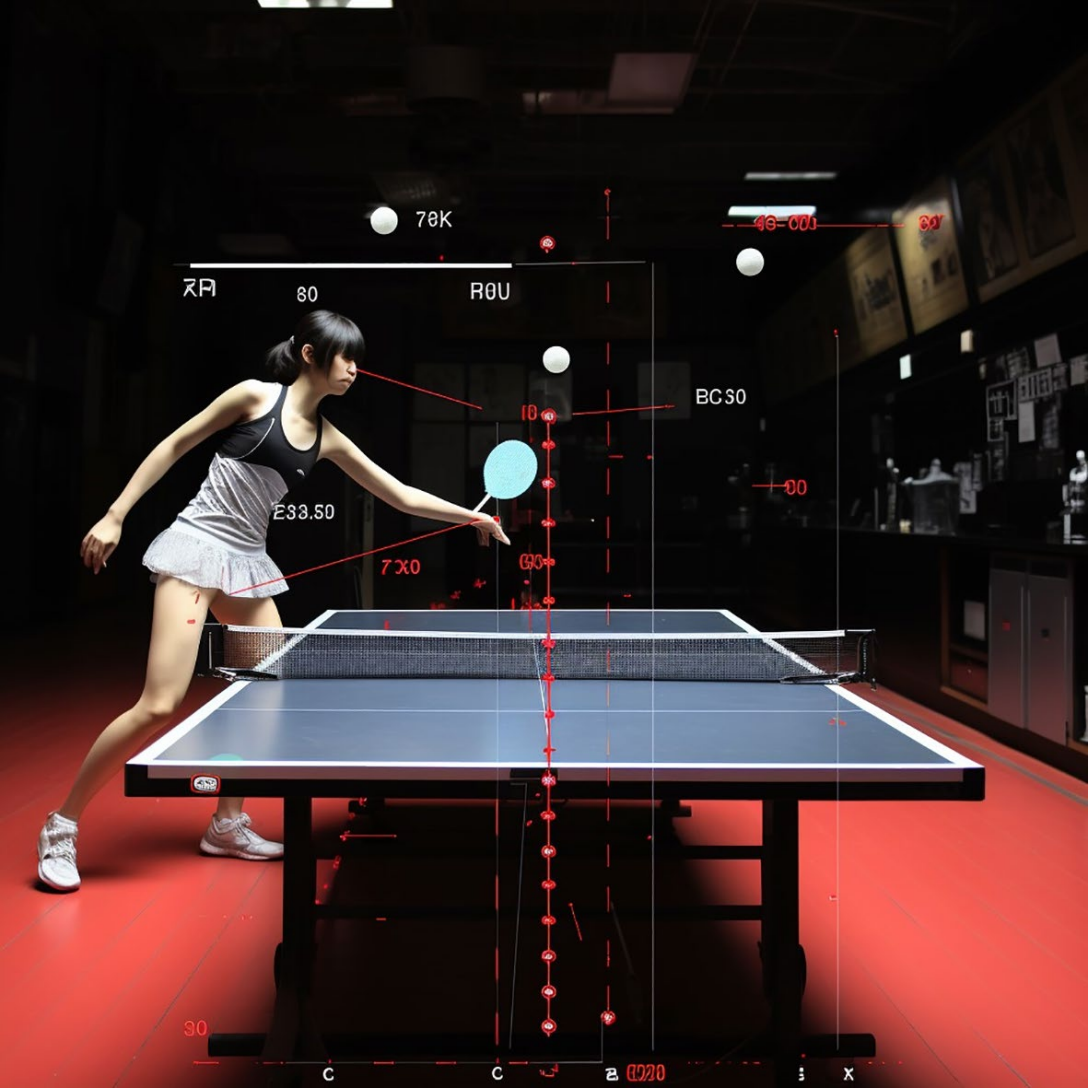
Table tennis TD effect of FAST R-CNN + FPN.

The table tennis TD effect of FAST R-CNN + FPN in this paper is shown in Fig. 9. The table tennis in Fig. 9 is accurately detected and calibrated, which shows the high accuracy of FAST R-CNN + FPN method in target location. The combination of FAST R-CNN and FPN enables the algorithm to capture the position information of table tennis more accurately, thus improving the accuracy of detection. It is worth noting that there are three table tennis balls in the image, and FAST R-CNN + FPN can effectively detect them at the same time. This highlights the superiority of this method in dealing with multi-TD scenes, and shows that the algorithm can still maintain high efficiency and accuracy if athletes need to pay attention to and deal with multiple balls at the same time in table tennis competition. Through testing in real scenes, the model can maintain high accuracy and robustness in complex and changeable competition environment, thus verifying the reliability of the model in practical application.

In the research process, the application of FAST R-CNN + FPN method in table tennis player training is investigated in detail. The performance of athletes in technical training with FAST R-CNN + FPN is measured and compared with other models. The effects of different training methods on athletes' technical level are shown in Table 5. By comparing the experimental results, the application of FAST R-CNN + FPN method in technical training has achieved more remarkable results than other models. Specifically, FAST R-CNN + FPN surpasses YOLOv7 and YOLOv8 in technical level improvement, accuracy improvement and effect evaluation. The technical level of FAST R-CNN + FPN is the highest, which is 15% and more significant than that of YOLOv7 (8%) and YOLOv8 (10%). In terms of accuracy improvement, FAST R-CNN + FPN has also made great progress, accounting for 10%, while YOLOv7 and YOLOv8 are 5% and 8% respectively. In the effect evaluation, FAST R-CNN + FPN gets the highest score, which is 4.5, surpassing the 3.5 score of YOLOv7 and the 4.0 score of YOLOv8. This result is attributed to the better application of deep learning and MFF technology in table tennis players' technical training by FAST R-CNN + FPN method. It combines the advantages of FAST R-CNN and the MFF of FPN, so that the model can capture the athletes' technical movements more comprehensively and accurately, thus achieving more remarkable results in improving the technical level and accuracy. In addition, the difference in scores also shows that FAST R-CNN + FPN is more positive in the overall effect. However, the performance of YOLOv7 and YOLOv8 in table tennis players' training is relatively weak, because they are not as flexible as FAST R-CNN + FPN in dealing with MFF, and they do not fully capture the key features of players' movements in the training process. The advantages of FAST R-CNN + FPN in deep learning and MFF technology may support its more significant improvement in table tennis players' technical training.

**Table 5:** Influence of different training methods on athletes' technical level.

Method	Technical level improvement (%)	Accuracy improvement (%)	Effect evaluation (score)
FAST R-CNN + FPN	15	10	4.5
YOLOv7^39^	8	5	3.5
YOLOv8^40^	10	8	4.0

### Discussion

The FAST R-CNN + FPN method proposed in this paper performs well in the task of table tennis TD, and the mAP value reaches 87.30%, which is superior to other algorithms (YOLOv4, Attention mechanism ConvNeXt, RetinaNet and R-CNN). This shows that the introduction of FPN and MFF further improves the performance of the algorithm through top-down deconvolution. YOLOv4 and Attention mechanism ConvNeXt are relatively poor in table tennis TD, and their average maps are 81.40% and 80.3% respectively. This is because their relatively simple feedforward structure does not make full use of different scale feature information, resulting in poor performance on table tennis targets. When comparing two different feature fusion networks, FPN and PANet, this paper points out that using PANet can improve the accuracy of DL TDA, but the reasoning time increases accordingly. Among them, the maximum mAP of YOLOv4 + PANet reaches 88.10%, while that of FAST R-CNN + FPN reaches 89.20%. Although PANet improves accuracy, the increase of reasoning time may be a trade-off. Compared with related scholars' research, Francies et al. (2022) used large-scale Pascal VOC dataset to test modern YOLO algorithms (YOLOv3, YOLOv4 and YOLOv5) in multi-class 3D TD and recognition. The final conclusion of the paper shows that YOLOv3 has achieved the highest recognition accuracy, with a mAP of 77%^41^. In this paper, FAST R-CNN + FPN achieves a higher mAP (87.30% vs. 77%) in table tennis TD, and performs better. Wu et al. (2022) optimized the structure of Mask R-CNN, helped tennis picking robot to perform target recognition, and improved its ability to acquire and analyze image information. The experimental results show that the improved algorithm based on Mask R-CNN achieves 92% accuracy in tennis recognition at the iteration level of 30 to 35, which is higher in accuracy and recognition distance than other tennis recognition algorithms^42^. Peng et al. (2023) proposed a heart rate measurement method based on face recognition. Through DL face recognition, this method has high computational efficiency and can effectively eliminate the influence of other external environmental factors. Video recording can be used to monitor athletes' heart rate changes in real time through face recognition and quantification of physiological parameters. The experimental results show that the heart rate error of video heart rate measurement algorithm is less than 3% in static state and less than 4% in post-exercise state, which can effectively measure psychological fluctuations^43^. The above research has jointly promoted the development of TD and biometric monitoring. The Fast R-CNN + FPN method proposed in this paper performs well in the task of table tennis TD, and has achieved remarkable advantages in accuracy and efficiency.

## Conclusions

A table tennis TD technique based on DL and MFF guidance is suggested in this paper. Through this method, the accuracy of ball detection in table tennis competition can be improved, the training process of athletes can be optimized, and the technical level can be improved. Based on FAST R-CNN, this method fuses different levels of feature information through MFF guidance, which improves the accuracy of TD. The experimental results show that this method has achieved remarkable advantages in table tennis TD, and its mAP reaches 87.3%, which is obviously superior to other TDAs and has higher robustness. Further analysis shows that the FAST R-CNN + FPN method performs better than other algorithms in table tennis TD. Compared with YOLOv4, Attention mechanism ConvNeXt, RetinaNet and R-CNN, this method adopts MFF to improve the performance of the algorithm. In the experiments of input images with different resolutions, the image with 416*416 resolution performs best in table tennis TD, and its mAP value is 89.40%. This may be because table tennis is relatively small, and high-resolution images may lead to too many pixels of the ball, which may affect the extraction of feature information and may lead to over-fitting problems. This paper has successfully applied the algorithm in the actual training process of table tennis players. By improving the training effect, the effectiveness and accuracy of technical training, FAST R-CNN + FPN has substantially improved the overall level of table tennis players. The success of this practical application provides strong support for the potential value of DL in sports training. Generally speaking, through the successful application in the table tennis TD task and the in-depth analysis of the factors affecting the performance of the algorithm, this paper provides useful experience and enlightenment for the research and practical application of DL in the field of sports.

However, there are still some shortcomings in this paper. First, this paper only explores the TD in table tennis competition, but does not conduct in-depth research on the optimization of table tennis technical training. Secondly, this paper only uses one dataset for experimental verification, and the dataset is relatively small, which lacks a comprehensive verification of the robustness of the algorithm. Finally, different algorithms may have different effects for different input resolutions, and may increase the calculation and memory occupation while improving the accuracy. In addition, although the introduction of FPN improves the performance of the algorithm, it is necessary to weigh the relationship between accuracy and reasoning time. Expanding the dataset, incorporating additional DL algorithms, and utilizing FF mechanisms of optimization algorithms in the future, exploring a specific network structure that is more suitable for table tennis TD, and considering more applications in complex scenes will all help to further improve TD accuracy and athletes' technical proficiency in table tennis match.

### Supplementary Information

Supplementary Information.

## Data Availability

All data generated or analysed during this study are included in this published article [and its supplementary information files].
