# Supplementary material for: Optimization of table tennis target detection algorithm guided by multi-scale feature fusion of deep learning
Source: Sci Rep. 2024 Jan 16;14:1401. doi: 10.1038/s41598-024-51865-3 (PMC10792085; doi:10.1038/s41598-024-51865-3)
Supplement: Supplementary file 1 — Supplementary Information. [file 41598_2024_51865_MOESM1_ESM.zip › Data packet/Code description.docx]

**Code description:**

The code is an object detection algorithm that includes basic steps for data loading, model construction, and training. The code uses the TensorFlow and Keras libraries and employs ResNet50 and FPN to build the object detection model. The “load_and_preprocess_data**”** function in the code is used to load and preprocess the dataset, ensuring that the data format is suitable for the model's input and label requirements. The code defines two models, FAST R-CNN and FPN, where the former is used for object classification and bounding box regression, and the latter is used for feature pyramid networks. The “compile**”** method is used to select optimizers, loss functions, and evaluation metrics for FAST R-CNN and FPN models. The “fit**”** method is employed to train the FAST R-CNN and FPN models, providing the loaded and preprocessed training data and labels. After training the models, the code preprocesses images by resizing them to 416×416 pixels, uses the models for prediction, and obtains the classification probabilities and bounding box coordinates of the objects. Object classification is performed based on the classification probabilities, and then bounding boxes are drawn on the images to identify the object's location.

To run the above code, we need to configure the following running environment:

Python environment: Python 3.8

NumPy numerical calculation library: installation command: pip install numpy

TensorFlow library: installation command: pip install tensorflow

Keras library: installation command: pip install keras
